# Association of biomarkers with health-related quality of life and history of stressors in myalgic encephalomyelitis/chronic fatigue syndrome patients

**DOI:** 10.1186/s12967-016-1010-x

**Published:** 2016-08-31

**Authors:** Emmanuel Fenouillet, Aude Vigouroux, Jean Guillaume Steinberg, Alexandre Chagvardieff, Frédérique Retornaz, Regis Guieu, Yves Jammes

**Affiliations:** 1 DS-ACI UMR MD2, Faculty of Medicine, Aix-Marseille University, Bd. Pierre Dramard, 13916 Marseille Cedex 20, France; 2 CNRS, Institut des Sciences Biologiques, Marseille, France; 3 Clinical Respiratory Physiology Laboratory, Nord Hospital, Marseille, France; 4 Emergency Unit, Nord Hospital, Marseille, France; 5 Internal Medicine Department, European Hospital, Marseille, France

**Keywords:** Myalgic encephalomyelitis/chronic fatigue syndrome, CD26, Muscle excitability, Quality of life, Oxidative stress

## Abstract

**Background:**

Myalgic encephalomyelitis chronic fatigue syndrome (ME/CFS) is a common debilitating disorder associated with an intense fatigue, a reduced physical activity, and an impaired quality of life. There are no established biological markerof the syndrome. The etiology is unknown and its pathogenesis appears to be multifactorial. Various stressors, including intense physical activity, severe infection, and emotional stress are reported in the medical history of ME/CFS patients which raises the question whether any physiological and biological abnormalities usually found in these patients could be indicative of the etiology and/or the quality-of-life impairment.

**Methods:**

Thirty-six patients and 11 age-matched healthy controls were recruited. The following variables that appear to address common symptoms of ME/CFS were studied here: (1) muscle fatigue during exercise has been investigated by monitoring the compound muscle action potential (M-wave); (2) the excessive oxidative stress response to exercise was measured via two plasma markers (thiobarbituric acid reactive substances: TBARS; reduced ascorbic-acid: RAA); (3) a potential inflammatory component was addressed via expression of CD26 on peripheral blood mononuclear cells; (4) quality-of-life impairment was assessed using the London Handicap Scale (LHS) and the Medical Outcome Study Short Form-36 (SF-36). The medical history of each patient, including the presence of stressors such as intense sports practice, severe acute infection and/or severe emotional stress was documented.

**Results:**

We observed that: (1) there were striking differences between cases and controls with regard to three biological variables: post-exercise M-wave, TBARS variations and CD26-expression at rest; (2) each of these three variables correlated with the other two; (3) abnormalities in the biomarkers associated with health-related quality of life: the LHS score was negatively correlated with the exercise-induced TBARS increase and positively correlated with CD26-expression while the pain component of SF-36 was negatively correlated with CD26-expression; (4) the TBARS increase and the M-wave decrease were the highest, and the CD26-expression level the lowest in patients who had been submitted to infectious stressors.

**Conclusion:**

In ME/CFS patients, severe alterations of the muscle excitability, the redox status, as well as the CD26-expression level are correlated with a marked impairment of the quality-of-life. They are particularly significant when infectious stressors are reported in the medical history.

## Background

As primarily defined by Fukuda [1], and in broad agreement with the latest case definition published in 2015 by the Institute of Medicine [2], the myalgic encephalomyelitis/chronic fatigue syndrome (ME/CFS) is currently described as a debilitating disorder characterized by an intense fatigue that is not improved by rest, and that is worsened by physical/mental activity. This clinical picture is usually associated with a marked loss of quality of life [1]. The definitions of ME/CFS do not use biological markers. Several body systems such as the muscular and nervous systems are affected in a context of neuro-immune alterations and chronic low-grade inflammation [3–6]. The etiology of ME/CFS is unknown, and its pathogenesis appears to be multifactorial, various stressors being repeatedly reported in the medical history of these patients, notably an intense sport practice, severe infections, and/or emotional stress [7, 8].

Whereas sport practice induces a moderate oxidative stress in healthy subjects [9], a strong exercise-induced production of reactive oxygen species (ROS) is found in ME/CFS patients [10–14]. The antioxidant response is also altered at rest, as shown by an increased level of the thiobarbituric acid reactive substances (TBARS: a marker of lipid peroxidation) and a decreased concentration of the reduced ascorbic acid marker (RAA: an endogenous antioxidant) [10, 13, 14]. Because the increased ROS production affects the muscle excitability [15, 16], the increased redox stress in ME/CFS may participate to the muscle fatigue as evidenced via an alteration of the compound evoked muscle action potential (M-wave) [10].

The significant dysregulation of the immune system found in ME/CFS patients combined increased levels of pro-inflammatory cytokines (e.g. interleukin-1, tumor necrosis factor-α), a natural killer cell cytokine production and mitogen-activated protein kinase [4–6]. A recent work also identified an association of immune abnormalities with the severity of ME/CFS symptoms and an altered quality of life [17]. These immunological abnormalities also involve a decreased expression of the T cell activation marker CD26 expressed on peripheral blood mononuclear cells (PBMC) [18]. This observation, together with the reports that CD26-expression is increased in patients with fibromyalgia [19] or a metabolic syndrome [20], indicates that CD26-expression changes are present in some muscular disorders.

Besides T-cell activation and inflammation control, CD26 may also be involved in ME/CFS by its endoproteolytic activity that controls the circulating levels of various effectors involved in anxiety, chronic pain and muscle metabolism. Thus, CD26 can influence the mood, pain and muscle components of ME/CFS [21–24]. The decrease in CD26-expression in ME/CFS may also be associated with an altered redox status [10–14], a relationship being reported between CD26-expression and redox disorders [25, 26].

Seeking help for the diagnosis of ME/CFS, previous studies have searched for a correlation between symptoms monitored using analogic scales of fatigue and markers, in particular, oxidative stress and immune markers [17, 27–29]. Other studies reported an alteration of the health-related quality-of-life in ME/CFS patients but did not examine the relationship with biological alterations [30–33].

As indicated above, current case definitions of ME/CFS are based on symptoms and do not use biomarkers. This raises the question of whether people with ME/CFS share any biological abnormalities. If they do the physiological and biological abnormalities found in these patients could become diagnostic markers.

## Methods

### Patients

The protocol was approved by the Ethics Committee of our institution (CPP Sud Mediterranée 1) and the study was carried out by the Code of Ethics of the World Medical Association (Declaration of Helsinki). The procedures have been conducted with the adequate understanding and written consent of the subjects (36 ME/CFS patients and 11 age-matched healthy controls). ME/CFS patients (mean age: 41 ± 6 years; mean weight: 66 ± 3 kg; 22 females) were recruited during this three-year long study (2014–2016) and satisfied the criteria defined by the Institute of Medicine of the National Academies [2]: (1) severe chronic fatigue for >6 consecutive months (36/36 cases); (2) a worsening of illness following increases in physical or cognitive activity (36/36 cases); (3) impairment of short-term memory or concentration (36/36 cases), (4) myalgia (24/36 cases); (5) unrefreshing sleep (21/36 cases). In most cases, the ME/CFS began suddenly (30/36), often with a “flu-like” illness (28/36). Exclusionary diagnoses are the medical and psychiatric causes of persistent bodily fatigue.

The data on ME/CFS patients were compared to those obtained in an age-matched control group of 11 healthy volunteers (age: 48 ± 5 years; weight: 71 ± 4 kg; 6 females; same socio-economic class). They consulted for a medical check-up and the medical practitioner did not report any symptoms of ME/CFS during the interview. The healthy volunteers underwent a systematic medical examination, including medical history, physical examination, and laboratory testing, which revealed no abnormalities. Median values of weight did not significantly differ between the ME/CFS and control groups. The characteristics of the subjects are collected in Table 1.

**Table 1:** Characteristics, redox status, CD26-expression and muscle fatigue parameters of ME/CFS patients and control subjects

	ME/CFS (n = 32)	Controls (n = 11)
Age (years)	42 ± 7	46 ± 5
Sex ratio (F/M)	24/12	6/5
ME/CFS duration (y)	5 ± 1	
At rest		
RAA/TBARS	91 ± 19*	129 ± 15
CD26-expression (AU)	2.72 + 0.09*	3.60 ± 0.09
End exercise		
VO2max (ml min^−1^ kg^−1^)	23 ± 1	26 ± 4
Δ M-wave (%)	−41 ± 8**	+8 ± 6
ΔTBARS (%)	+50 ± 10**	+15 ± 7

At rest: RAA/TBARS: ratio of antioxidant response (Reduced ascorbic acid, RAA) to lipid peroxidation (Thiobarbituric acid reactive substances, TBARS); CD26-expression measured via DPP-IV activity (AU: arbitrary unit)

Exercise: VO2 max (maximal oxygen uptake); ΔM-wave amplitude: (maximal decrease in M-wave amplitude evoked in the *rectus femoris*); ΔTBARS (maximal increase in TBARS post-exercise) (Mean + SEM; * p < 0.05; ** p < 0.01)

Considering their medical history, four groups of patients were retrospectively constituted: (1) patients reporting an intense sport practice (>6 h/week) (n = 10), (2) patients reporting a severe acute infection (peritonitis, sepsis, avian influenza…) diagnosed within the 3–7 month period preceding the onset of ME/CFS, (n = 7), (3) patients reporting a severe emotional stress (difficult divorce, death of a child…) (n = 11), or (4) patients combining severe infection and emotional stress (n = 8).

### Psychometrical indices of health-related quality of life

The ME/CFS health-related quality of life was evaluated using two questionnaires: the validated French versions of the London Handicap Scale (LHS) [34] as well as the Medical Outcome Study Short Form-36 (SF-36) [35], this latter being divided into three item groups (physical function, bodily pain, and vitality).

### Biochemical redox variables

Blood from an antecubital vein was collected on lithium heparinate at rest and at the end of the cycling exercise. An aliquot sample was used to measure TBARS and reduced ascorbic-acid (RAA) according to methods previously published [8, 10, 11, 36] and originally described by Uchiyama and Mihara [37] and Maickel [38], respectively.

### CD26 peptidase activity

PBMCs were isolated from the blood using the Vacutainer CPT system (Becton–Dickinson). As previously described [19, 23], PBMCs (1.10^6^ cells) were then incubated for 60 min at 37 °C in 75 mM glycine buffer pH 8.7 with three mM Gly-Pro-*p*-nitroanilide, a colorimetric substrate of the peptidase CD26. The signal background was determined by incubation in acetate buffer pH 5, a condition in which DPPIV is inactive. Optical density values were determined (405 nm).

### M-wave recordings and analyses

As previously reported [10], bipolar (30 mm center-to-center) Ag–AgCl surface electrodes (Dantec, 13 L 20) were used to measure EMG voltage from one *rectus femoris* muscle. The electrodes were placed between the motor point and the proximal tendon. The EMG signal was amplified (Nihon Kohden, Tokyo, Japan) in the 10–2000 Hz range. Compound muscle action potentials (M-waves) were evoked in the *rectus femoris* by direct stimulation using a monopolar technique and a constant-current neurostimulator (S88 model Grass, Quincy, MA) that delivered supra-maximal shocks with 0.1 ms rectangular pulses. The EMG signal was fed to an oscilloscope (model DSO 400, Gould), permitting to average M-waves from 8 successive potentials and to calculate the peak M-wave amplitude. The maximal changes in M-wave amplitude at the end of exercise (ΔM-wave; %) was expressed versus rest value.

### Maximal cycling exercise

After a 30-min rest period, each subject performed an incremental exercise test on an electrically braked cycle ergometer (Ergometrics ER 800, Jaeger) [8, 10, 11, 36]. Throughout the incremental exercise trial, the software averaged each variable for ten consecutive seconds. The maximal VO_2_ value (VO_2_max) was measured when the subject had reached his/her predicted maximal heart rate. M-wave recordings and blood samplings for biochemical analyses were performed at the end of the exercise (VO_2_max, and at the 5th min post-exercise).

### Statistical analysis

Data are presented as mean ± standard error of means (SEM). A two-way ANOVA was performed to compare the baseline levels of the biochemical markers between ME/CFS patients and controls. The least square regression analysis was used to compare CD26-expression, TBARS (at rest and post-exercise), M-wave amplitude variations and LHS/MOS SF-36 data. The significance was considered when P < 0.05.

## Results

### Biochemical variables and muscle excitability at rest and at VO_2_max

Table 1 shows the significant biochemical differences observed between ME/CFS patients and controls at rest: the RAA/TBARS ratio and the expression of surface CD26 per PBMC were lower in the patients.

Exercise-induced changes in M-wave amplitude (ΔM-wave) were significantly higher in patients than in controls (Table 1). A significant increase in TBARS post-exercise was found in patients only. Because the duration of the exercise test (10–12 min) is well below the time needed for the de novo synthesis and cell surface expression of CD26 [39], we did not examine in all patients whether the cycling exercise could affect CD26 expression (we addressed the situation in 10 patients and did not find any differences).

Together, the data obtained at rest and VO_2_max show that the redox status, CD26-expression, and muscle excitability were altered in ME/CFS.

**Fig. 1:**
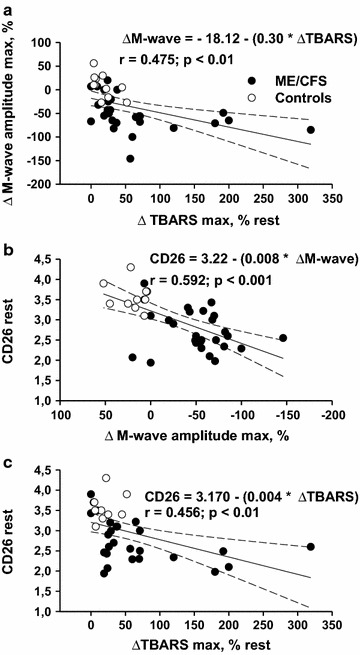
M-wave, exercise-induced redox stress and CD26-expression. Correlation between the decrease in M-wave amplitude post-exercise (ΔM-wave) and the maximal increase in TBARS level induced by exercise (ΔTBARS) (percent of its resting level; **a**) Correlation between ΔM-wave and CD26-expression at rest (**b**). Correlation between ΔTBARS and CD26-expression at rest (**c**). Data in ME/CFS patients (n = 36) and control healthy subjects (n = 11) are reported. Each point could correspond to different individuals. Least square linear regression with 95 % confidence intervals is shown

**Fig. 2:**
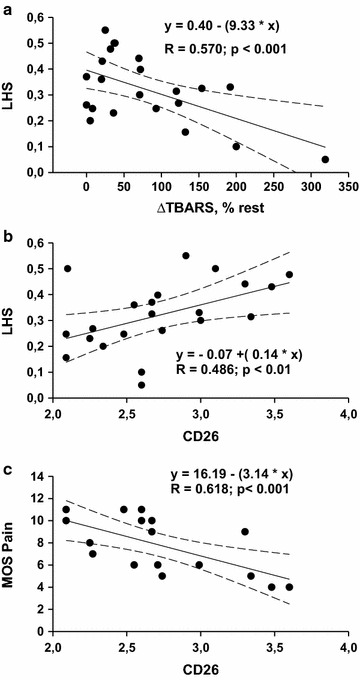
Quality-of-life, exercise-induced redox stress and CD26-expression. Correlation between the London Handicap Scale (LHS) score and ΔTBARS (**a**) or CD26-expression (**b**). Correlation between the pain component of MOS SF-36 and CD26-expression (**c**). Only data in ME/CFS patients (n = 36) are shown. Each point could correspond to different individuals. Least square linear regression with 95 % confidence intervals is shown

**Fig. 3:**
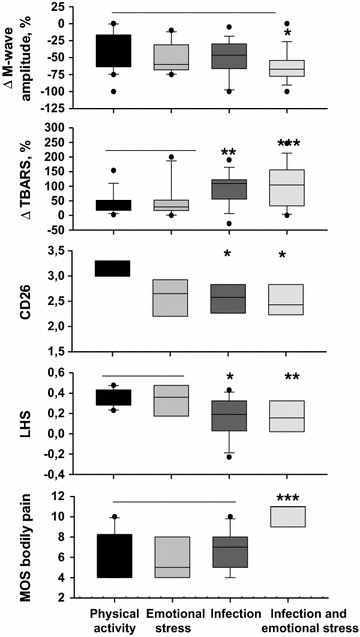
Effects of stressors on M-wave, TBARS level, CD26 expression and quality of life scores. Data are represented by *box* plots with 5th and 95th percentiles. Asterisks indicate that values significantly differ from those measured in groups of patients with horizontal bar on *top* (*p < 0.05; **p < 0.01; ***p < 0.001)

When we examined whether these characteristics are associated, we found (1) a negative correlation between ΔM-wave and ΔTBARS (Fig. 1a), (2) a positive correlation between ΔM-wave and CD26-expression (Fig. 1b), and (3) a negative correlation between ΔTBARS and CD26-expression (Fig. 1c). We found no correlation at rest between the TBARS level, RAA/TBARS ratio and CD26-expression.

### Relationship between biological markers and health-related quality of life

In the patients’ population, the scores of health-related quality-of-life were plotted according to the level of ΔTBARS or CD26-expression. The LHS score was negatively correlated with ΔTBARS (Fig. 2a) and positively correlated with CD26-expression (Fig. 2b). The pain component of the SF-36 questionnaire was negatively correlated with CD26-expression (Fig. 2c).

### Relationship between biological markers and stressors

When the four groups of patients were constituted considering their medical history, we observed that: (1) the M-wave was found to be significantly reduced only when both infection and emotional stressors were combined; (2) ΔTBARS levels and CD26 expression were markedly altered in patients who had a severe infection, regardless of the presence of a severe emotional stress; (3) the LHS score was markedly low in patients who had a severe infection regardless of the presence of a severe emotional stress, while the score of the pain component of SF-36 was high only when both infection and emotional stressors were combined. Figure 3 collects these data. These results support the importance of infectious stressors in the behavior of the variables we monitored.

## Discussion

In ME/CFS patients, we found that (1) the changes in the M-wave amplitude post-exercise, the alterations of the redox status induced by muscle exercise, and the CD26-expression level are correlated; (2) the LHS score was correlated to the alterations of the redox status induced by exercise and CD26-expression; (3) the pain component of MOS SF-36 was correlated to the CD26-expression level. These variables were mainly altered in patients with a history of infectious disease.

The oxidative stress refers to an imbalance in the pro- and anti-oxidant status in favour of the former. In healthy subjects, this situation is common in skeletal muscle following exercise because the muscle anti-oxidant defences are weak [9, 36]. In ME/CFS patients, the present work and others reported increased levels of blood markers of oxidative stress (here, TBARS) and a decreased antioxidant defense (here, RAA), a situation that promotes an oxidative stress, and eventually affects the muscle membrane excitability [10–16]. The correlation found here between the level of oxidative stress and the M-wave alteration is consistent with these data.

Our work also reports the correlation of the redox markers with the LHS score of quality-of- life. This relationship is in agreement with studies that examined the impact of an altered oxidant-antioxidant status in ME/CFS and found that the resting blood levels of malondialdehyde [27] and TBARS [28] are associated with variations in cognitive symptoms and sleep disturbances whereas the total symptom score, joint pain, and postexertional malaise correlate with the isoprostane levels [29].

Regarding CD26, we found that its expression was decreased in ME/CFS patients. This observation supports a previous report [18] but contrasts with a study on ME/CFS patients examined after Giardia infection [40]. The CD26 protease activity controls the circulating level of several mediators of pain and mood [21–24]. Because mood changes and myalgia often occur in ME/CFS [1, 2], concentrations of CD26 peptide substrates were addressed in patients, and discrepant conclusions were reached regarding Neuropeptide Y and Substance-P [41, 42]. Here, we found that the scores reflecting the quality-of-life are correlated with CD26-expression. This result is in line with the present observations that myalgia was often reported by our patients (24/36 patients), impacting their quality-of-life scores. We also observed that the CD26 expression level decreased, which may elevate the circulating level of pro-nociceptive peptides and promote myalgia.

Recently, two studies have investigated the CD26-expression level in a context of redox alterations. In acute lymphoblastic leukemia, the CD26 level is correlated with the myeloperoxidase activity, glutathione-s-transferase activity and xanthine oxidase activity [25]. Moreover, a long term loss of CD26-expression was shown to increase the capability to protect against the oxidative stress [26]. The low CD26-expression level here reported in ME/CFS may constitute therefore an adaptive response to the chronic redox imbalance found in ME/CFS patients [10–14 and the present study], this hypothesis being supported by the correlation we found between the CD26 activity and the redox status.

Finally, when patients were sorted according to the presence of infection/intense physical activity/emotional stress in their medical history, we observed that the subjects who reported episode(s) of severe infection had levels of biomarkers and scores of quality-of-life that were more altered compared with those measured in patients who did not report severe infections. These results further support the role of infectious stressors in ME/CFS [7, 8].

We believe that this study has three main limitations: (1) we did not properly quantify the emotional stress because only the presence of a major stressful life event was considered; (2) in order to address the effect of CD26 activity on its peptide substrates, we undertook to measure the circulating levels of neuropeptide Y and substance-P in patients but we did not detect correlation with the CD26-expression level, which may be explained by the fact that only local concentrations of pain and mood mediators act in vivo,their sampling in situ being impossible; (3) as in previous ME/CFS studies [8, 10–14], the finding of an altered redox balance in our subjects was only based on measurements of TBARS (which quantifies the level of membrane lipoperoxydes resulting from an excessive ROS production) and of the reduced ascorbic acid (a major plasma antioxidant). Thus, the effective enhancement of intracellular ROS production in ME/CFS cannot be evaluated first because it would have required muscle biopsies and second because it is counterbalanced by the intracellular antioxidants that are not explored in the present study.

## Conclusion

In accordance with a recent report stating that severe ME/CFS patients differover time from moderate ME/CFS patients and express significant immune abnormalities [17], our results indicate that the simultaneous monitoring of the muscle function, redox response, and CD26-expression could contribute, together with the health status scales, to identifying ME/CFS and to assessing its severity. These results may also help to distinguish ME/CFS from fibromyalgia, because the CD26 activity on PBMC increases in fibromyalgia [19] whereas it decreases in CFS patients.

## Abbreviations

DPP-IV: dipeptidyl peptidase-IV
EMG: electromyography
ΔM-wave: maximal decrease in M-wave amplitude
Δ TBARS: exercise-induced increase in TBARS level vs rest level
LHS: London Handicap Scale
ME/CFS: myalgic encephalomyelitis/chronic fatigue syndrome
MOS SF-36: Medical Outcome Study Short Form-36
M-wave: compound muscle action potentials
PBMC: peripheral blood mononuclear cells
RAA: reduced ascorbic acid
TBARS: thiobarbituric acid reactive substances
TCA: trichloracetic acid
VO_2_max: maximal oxygen uptake
